# Acute Disseminated Encephalomyelitis Presenting as Bilateral Ptosis in a Sri Lankan Child

**DOI:** 10.1155/2022/5492155

**Published:** 2022-02-08

**Authors:** Ishara Kumarasiri, Ruwan Samararathna, Udara Sandakelum, Oshanie Muthukumarana, Reha Balasubramaniam, Sachith Mettananda

**Affiliations:** ^1^University Paediatrics Unit, Colombo North Teaching Hospital, Ragama 11010, Sri Lanka; ^2^Department of Paediatrics, Faculty of Medicine, University of Kelaniya, Thalagolla Road, Ragama 11010, Sri Lanka

## Abstract

**Introduction:**

Acute disseminated encephalomyelitis is a rare inflammatory demyelinating disease characterized by acute onset polyfocal neurological deficits associated with encephalopathy. It commonly presents with fever, meningism, seizures, ataxia, motor deficits, and bladder dysfunction. Although cranial neuropathies, including optic neuritis and facial nerve palsies, have previously been reported, children presenting with bilateral ptosis is extremely rare. Here, we report a 3-year-old child with acute disseminated encephalomyelitis presenting with acute onset bilateral ptosis due to involvement of the single central levator subnucleus of the oculomotor nerve. *Case Presentation*. A 3-year-old Sri Lankan boy presented with drooping of the upper eyelids for three days and unsteady gait for two days. He did not have seizures, blurring of vision, limb weakness, swallowing or breathing difficulties, or bladder dysfunction. On examination, he had bilateral ptosis, gait ataxia, and dysmetria. His vision, eye movements, and examination of other cranial nerves were normal. MRI brain revealed high signal intensities involving the subcortical white matter of parietal and occipital lobes, midbrain in the area of single central levator subnucleus of the oculomotor nerve, cerebellar vermis, and right cerebellar hemisphere. Based on the clinical features suggesting polyfocal neurological involvement of the midbrain and cerebellum and characteristic MRI findings, the diagnosis of acute disseminated encephalomyelitis was made. He responded well and rapidly to high-dose intravenous methylprednisolone and showed a complete clinical and radiological recovery.

**Conclusion:**

This case report describes a rare presentation of acute disseminated encephalomyelitis, bilateral ptosis due to involvement of the single central levator subnucleus of the oculomotor nerve. It highlights that the presenting manifestations of acute disseminated encephalomyelitis can be subtle and vary; however, timely diagnosis and treatment result in complete recovery.

## 1. Introduction

Acute disseminated encephalomyelitis (ADEM) is a rare inflammatory demyelinating disease of the central nervous system characterized by acute onset polyfocal neurological deficits associated with encephalopathy [1]. The common presentations include fever, headache, meningism, seizures, ataxia, motor and sensory deficits in limbs, and bladder dysfunction due to myelopathy [2]. Cranial neuropathies, including optic neuritis and facial nerve palsies, are reported; however, bilateral ptosis is rare. Here, we report a 3-year-old child with ADEM presenting with acute onset bilateral ptosis due to involvement of the single central levator subnucleus of the oculomotor nerve.

## 2. Case presentation

A 3-year-old previously healthy Sri Lankan boy presented with drooping of the upper eyelids for three days and unsteady gait with sleepiness and reduced activity for two days. He did not have seizures, blurring of vision, limb weakness, swallowing or breathing difficulties, or bladder dysfunction. He was born to nonconsanguineous, healthy parents, had a normal perinatal period, and was developmentally age-appropriate. There was no recent history of vaccination, febrile illnesses, snake bite, or behavioural changes before the onset of symptoms.

On examination, he was conscious and responsive with a GCS of 15/15. He had bilateral ptosis; however, his vision, eye movements, size of the pupils, pupillary light reflex, fundoscopy, and examination of other cranial nerves were normal. He walked with a broad-based ataxic gait and had dysmetria with positive finger nose test bilaterally. He did not have dysarthria, nystagmus, involuntary movements, or Romberg sign. Limb examination revealed normal tone and grade five muscle power in all four limbs and normal upper limb reflexes. The knee and ankle jerks were easily elicitable, and plantar responses were flexor bilaterally. There was no muscle fatiguability, sensory involvement, or signs of meningism. The examination of cardiovascular and respiratory systems was normal.

**Figure 1:**
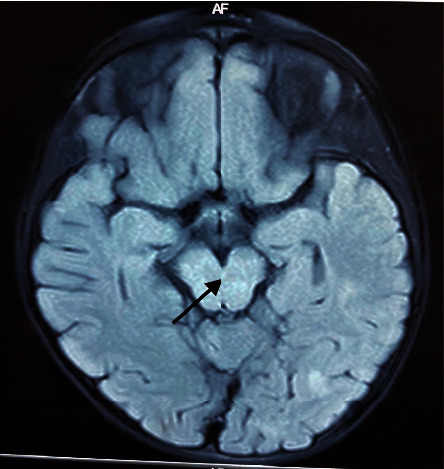
MRI brain during the acute stage showing high signal intensities in the midbrain at the site of single central levator subnucleus of the oculomotor nerve (arrow).

His complete blood count, C-reactive protein, serum electrolytes, and calcium were normal, and the ESR was 22 mm/1^st^ hour. The cerebrospinal fluid (CSF) examination revealed lymphocytic pleocytosis (polymorphs 2/mm^3^ and lymphocytes 20/mm^3^) with elevated proteins (55 mg/dL); however, the CSF was negative for oligoclonal bands. EEG and noncontrast CT brain were normal. MRI brain revealed high signal intensities involving the subcortical white matter of parietal and occipital lobes, midbrain, cerebellar vermis, right cerebellar hemisphere, and right caudate and lentiform nuclei without diffusion restriction or contrast enhancement (Figure 1). Myelin oligodendrocyte glycoprotein (MAG) antibody test was not performed due to unavailability.

**Figure 2:**
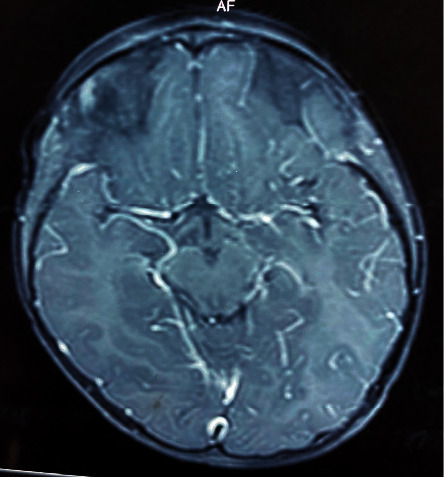
MRI brain performed three months after treatment, showing marked improvement in signal intensity changes in the midbrain.

Based on the clinical features suggesting polyfocal neurological involvement of the midbrain and cerebellum and characteristic MRI findings, the diagnosis of ADEM was made. He was treated with high dose (30 mg/kg) intravenous methylprednisolone for seven days and tapering course of oral steroids for four weeks. He demonstrated a gradual improvement in ptosis and ataxia and had complete clinical recovery after four weeks. The MRI brain performed after three months showed a marked reduction in the white matter and brainstem signal intensity abnormalities with minimal residual changes (Figure 2).

## 3. Discussion

ADEM is a rare acute demyelinating disorder of the central nervous system which has an annual incidence of 0.2–0.4 per 100,000 children [3]. It is characterized by multifocal white matter involvement in the brain and spinal cord [2]. The existing evidence suggests that ADEM results from a transient autoimmune response against myelin or other autoantigens, through molecular mimicry or by nonspecific activation of autoreactive T cell clone [4]. Although the disease can occur at any age, it is commonly reported in children aged between 5 and 8 years with a slight male preponderance [5].

The common neurological manifestations of ADEM are altered sensorium, meningism, seizures, quadriplegia, paraplegia, bladder involvement, dystonia, choreiform movements, nystagmus, ataxia, dysarthria, and neuropsychiatric manifestations [6]. Multiple cranial nerve involvement is also reported. The facial nerve is the most common cranial nerve to be involved. In addition, diplopia and ophthalmoplegia are reported as rare, presenting features of ADEM [7].

The most unusual feature of our patient is that he presented with bilateral ptosis without ophthalmoplegia or other cranial nerve palsies. The pathophysiological mechanisms causing ptosis include lesions of the oculomotor nerve and its nucleus and autonomic disturbance due to Horner syndrome. Isolated bilateral complete ptosis without paralysis of external ocular muscles could only be explained by the involvement of central levator subnucleus of the oculomotor nerve. This is because the innervation of both eyelids is through nerve fibres originating from this single central brainstem subnucleus. The MRI findings of brainstem involvement at the site of this subnucleus in our child confirm the causal relationships between demyelination and clinical features.

The differential diagnoses of our patient were autoimmune encephalitis, neuromyelitis optica, and the first episode of multiple sclerosis. The short duration of the illness before admission, absence of areflexia and motor weakness, simultaneous widespread multifocal involvement on MRI brain, and the dramatic response to steroids favour the diagnosis of ADEM. CSF studies in ADEM are often normal or can exhibit pleocytosis with lymphocytic predominance with elevated protein level. However, true positivity for oligoclonal bands is rare [8].

In conclusion, we report a child with ADEM presenting with bilateral ptosis, which is an unusual presentation of the disease. This case report highlights presenting manifestations of ADEM can be subtle and vary; however, timely diagnosis and treatment result in complete and rapid recovery.

## Data Availability

All data relevant to the case are included in the case report.
